# Circulating Tumor DNA in Gastric and Gastroesophageal Junction Cancer

**DOI:** 10.3390/curroncol29030120

**Published:** 2022-02-25

**Authors:** Lisa Paschold, Mascha Binder

**Affiliations:** Department of Internal Medicine IV, Oncology/Hematology, Martin-Luther-University Halle-Wittenberg, 06120 Halle (Saale), Germany; lisa.paschold@uk-halle.de

**Keywords:** gastric adenocarcinoma, gastroesophageal junction cancer, cell-free DNA (cfDNA), liquid biopsy, monitoring, resistance, prognostication

## Abstract

Tumor cells shed DNA into the plasma. “Liquid biopsy” analysis of mutations or other genomic alterations in circulating cell-free DNA (cfDNA) may provide us with a tool to detect minimal residual cancer, comprehensively profile the genomic tumor landscape in search of druggable targets, and monitor cancers non-invasively over time for treatment failure or emerging treatment-resistant tumor subclones. While liquid biopsies have not yet entered routine clinical management in patients with gastric and gastroesophageal junction cancers, this group of diseases may benefit from such advanced diagnostic tools due to their pronounced genetic spatiotemporal heterogeneity and limitations in imaging sensitivity. Moreover, as the armamentarium of targeted treatment approaches and immunotherapies expands, cfDNA analyses may reveal their utility not only as a biomarker of response but also for precision monitoring. In this review, we discuss the different applications of cfDNA analyses in patients with gastric and gastroesophageal junction cancer and the technical challenges that such liquid biopsies have yet to overcome.

## 1. Introduction

Despite overall declining incidences, gastric and gastroesophageal junction cancers—which are mostly adenocarcinomas—remain common malignancies. This group of diseases is the third to fourth most common cause of cancer death worldwide [1].

In the non-metastatic setting, the prognosis has improved through perioperative chemotherapy with fluoropyrimidines, platinum, and taxanes [2]. In non-resectable or metastatic tumors, systemic palliative chemotherapy is based on fluoropyrimidines, platinum, taxanes, irinotecan, and, in the refractory situation, trifluridine/tipiracil [3,4]. In second-line treatment, the vascular endothelial growth factor receptor-2 (VEGFR2) inhibitor ramucirumab is approved as monotherapy or in combination with paclitaxel [5,6]. Tumors with overexpression of human epidermal growth factor 2 (HER2) can be successfully treated with the anti-HER2 monoclonal antibody trastuzumab (plus chemotherapy), which has significantly improved the prognosis of this patient subgroup [7]. In addition, the immune checkpoint inhibitors nivolumab and pembrolizumab have recently been included in first-line treatment for patients with PD-L1-positive tumors in combination with chemotherapy [8,9]. Most recently, the fibroblast growth factor receptor (FGFR) pathway has received increased attention as a promising target and clinical trials are currently testing its inhibition [10,11,12,13].

Despite these advances, a number of challenges remain. These include the reliable detection of actionable lesions for targeted therapies and the sensitive monitoring of disease and resistance to open up avenues of precision targeting to more patients.

In this review, we discuss our current understanding of gastric cancer genetics and the role of cfDNA genomic analyses as an emerging diagnostic tool in precision medicine of this spectrum of tumors. Since cfDNA is extracted from blood plasma, this so-called liquid biopsy is a non-invasive technique and as such opens up the possibility for tight monitoring intervals of otherwise hardly accessible solid tumors. In metastatic settings or cases with a heterogeneous tumor architecture, such as in gastric and gastroesophageal junction cancer, liquid biopsy has the advantage of more broadly reflecting all tumor subclones than conventional biopsies. This increases the chances that relevant mutations and resistance mechanisms are detected. The work summarized in this review shows how liquid biopsies may promote our diagnostic abilities to improve precision diagnostics, tumor and resistance monitoring, andearly relapse detection, thereby refining our care for patients with gastric and gastroesophageal junction cancers.

## 2. Genetics of Gastric and Gastroesophageal Junction Adenocarcinoma

### 2.1. Frequent Genetic Alterations and Therapeutic Implications

Genomic analyses have shown that gastric and gastroesophageal junction adenocarcinomas have highly complex genomes characterized by both mutations and somatic copy number alterations in key genes [14,15,16,17,18]. These genetic alterations largely overlap in gastric and gastroesophageal junction cancer.

One of the therapeutically most important genes is ERBB2. The ERBB2 gene is a proto-oncogene whose protein product HER2 is a membrane-bound tyrosine kinase receptor that generates proliferative and anti-apoptotic signals when activated and is an important driver in tumor development and progression [19]. In gastric and gastroesophageal junction cancer, the percentage of HER2-positive tumors is similar to that in breast cancer, even if other criteria of positivity are applied [20]. Approximately 12–18% of gastric carcinomas and 24–32% of gastroesophageal junction cancers are HER2 positive [21]. While HER2 positivity represents a negative prognostic marker in breast cancer, it is not an independent negative prognostic marker in the spectrum of gastric and gastroesophageal junction cancers. The targeting of HER2 by trastuzumab in the palliative treatment setting of gastric and gastroesophageal junction cancer has significantly improved progression-free and overall survival in this subset [7].

Another gene that is amplified in up to one-third of patients and that has lately receiving increased attention is the fibroblast growth factor receptor (FGFR). This receptor is implicated in tumorigenesis and chemoresistance in many tumors [22]. Gen amplification is of clinical importance due to the development of anti-FGFR2-drug-targeting strategies, such as the monoclonal antibody bemarituzumab, which is in late-phase clinical development [23,24,25].

In routine clinical practice, HER2 and FGFR2 overexpression is determined by immunohistochemistry staining (IHC) or fluorescence in situ hybridization (FISH) on tumor slides. In both cases, tumor material obtained from a resection or biopsy is needed. The detection of copy number alterations (CNAs) from liquid biopsy would circumvent the need for surgical procedures in cases where not enough primary material is acquired or in the case of re-testings during the course of treatment. CNAs can reliably be detected from cfDNA [26] and were identified in gastric cancer for ERBB2 and FGFR2 by NGS [27,28] or digital droplet PCR (ddPCR) [29,30]. Thus, the profiling cfDNA can aid in identifying suitable treatment modalities for patients.

### 2.2. Genetic Classification

In the context of The Cancer Genome Atlas research project (TCGA) [14], gastric and gastroesophageal junction adenocarcinomas were categorized into four molecular subtypes defined as EBV-positive (9%), microsatellite instability (MSI) high (21%), genomically stable (20%), and chromosomal instability (CIN) (50%). In addition to its high EBV burden, the first subtype shows extensive DNA promoter hypermethylation, including that of CDKN2A. In addition, these tumors often display high PD-L1 expression. This is partly due to amplification of its gene CD274 [31]. The MSI group shows mutations in major histocompatibility genes, including beta-2 microglobulin (B2M), which may reduce antigen presentation in these highly mutated cancers. The genomically stable subtype essentially includes cancers of the Lauren diffuse subtype and the CIN group is characterized by extensive SCNA.

While, so far, the TCGA subtypes remain without clear predictive utility, they should be taken into consideration in future clinical trials with targeted agents and combination approaches. It is noteworthy that specific mutations are overrepresented in the four subtypes, such as CDH1 and RHOA mutations in genomically stable tumors. A high PIK3CA mutation rate was observed in the EBV and MSI groups, which may be of future therapeutic relevance.

The correlation of these subtypes with distinguishable genetic patterns opens up the possibility to classify gastric and gastroesophageal junction adenocarcinomas via sequencing of cfDNA, which is suitable to detect mutations, copy number variations, and aberrant DNA methylation, and may ultimately aid in prognostic assessment and treatment decision making.

### 2.3. Genetic Heterogeneity

Many solid tumors show spatiotemporal heterogeneity. In gastric and gastroesophageal cancers, this heterogeneity is pronounced [32,33,34]. Very convincingly, this was demonstrated by a multiregional sequencing study performed in the context of a neoadjuvant treatment protocol [35]. In this trial, whole exome sequencing (WES) was performed on 8 patients, including more than 40 tumor regions, prior to and after neoadjuvant chemotherapy. Interestingly, more than half of all mutations were heterogeneously present in different tumor subclones. Therefore, the sequencing of single biopsy specimens may greatly underestimate the mutational burden of the disease. From a biological perspective, the finding of a poor response to neoadjuvant treatment in tumors with high genomic heterogeneity was expected. From a diagnostic and monitoring perspective, the subclonal heterogeneity of gastric and gastroesophageal cancer indicates a role for liquid biopsies to more faithfully map the subclonal mutational landscape in individual patients.

## 3. Liquid Biopsy by Genetic Analysis of Circulating cfDNA in Gastric Cancer

### 3.1. Liquid Biopsy Analysis of cfDNA in Patients with Solid Cancers

cfDNA is released from somatic tissue into the blood stream after cell death. Cell-free DNA fragments deriving from apoptotic cells are usually only ~180 bp long [36], which has to be taken into account when designing analysis strategies of cfDNA. This process constitutively happens in healthy individuals, e.g., depending on tissue remodeling, leading to varying concentrations of cfDNA in plasma. Cancer patients, however, show increased amounts of plasma cfDNA [37,38]. Of these, patients with metastatic disease or advanced disease stages yield the highest amounts of cfDNA [38,39]. An important fraction of this represents the circulating tumor DNA that is defined by mutations or other genomic aberrations [36,40]. Analysis of cfDNA may provide information on the genomic landscape of a given tumor, including gene mutations, SCNA, chromosomal rearrangements, and methylation patterns. It may reflect both the primary tumor and the metastatic sites [41]. As such, it is well suited for the genetic characterization of tumors with a high degree of subclonal heterogeneity [42,43]. The detection of cfDNA with tumor-specific genetic aberrations after treatment with curative intent defines minimal residual disease (MRD). MRD may give rise to clinical relapse and cancer progression in the course. Liquid biopsy monitoring approaches after surgery or other forms of definitive treatment have been shown to enable the detection of disease relapse many months prior to clinical progression [44,45]. Furthermore, in the palliative treatment setting, tumor dynamics may be monitored non-invasively by quantifying tumor cfDNA over time [46,47,48]. Such serial cfDNA analyses can also provide insights into the molecular evolution of the clonal tumor makeup, with implications for therapy at progression [49,50,51].

### 3.2. Technical Challenges in Liquid Biopsy Analyses of cfDNA

**Figure 1:**
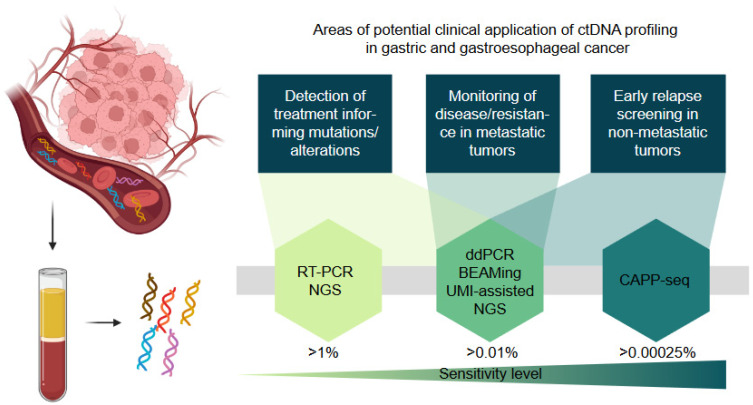
Schematic presentation of the potential applications of cfDNA profiling in gastric and gastroesophageal cancer. The sensitivity level indicates the minimal allele frequency (%) of a mutation that can be detected with the respective method. RT-PCR = real-time PCR; NGS = next-generation sequencing; ddPCR = digital droplet PCR; BEAMing = beads, emulsion, amplification, magnetics digital PCR technique; UMI = unique molecular identifier, CAPP-seq = cancer personalized profiling by deep sequencing.

There are many challenges in the analysis of liquid biopsies in cancer patients as recently reviewed in [52]. These include preanalytical problems associated with the unspecific lysis of blood cells releasing large quantities of unmutated DNA if no adequate asservation technique and stabilizers are used. Moreover, very low levels of tumor cfDNA in early cancer stages dictate low sensitivity of detection. Additional pitfalls arise from the potential detection of mutations that derive from clonal hematopoiesis or uncommon heterozygous single nucleotide polymorphisms that may be erroneously interpreted as tumor specific [32]. The majority of liquid biopsy studies that investigate the prognostic relevance of this biomarker and its utility for monitoring quantify the percentage of tumor-derived cfDNA by sequencing (e.g., NGS, modified NGS, CAPP-seq) or individual mutation detection (e.g., RT PCR, ddPCR, BEAMing). These technologies have different sensitivity levels as shown in Figure 1.

Due to the stage-specific amounts of tumor-derived cfDNA, caution must be exercised when selecting a technology platform with adequate sensitivity for the respective research question. A general problem of all mutation-based quantification strategies consists in inadequate representation of the whole tumor mass by single mutations in cancers with pronounced subclonal genetic heterogeneity. For prognostication and monitoring, a number of studies have, therefore, used quantification of plain cfDNA concentrations per plasma volume based on the observation that cfDNA concentrations correlate with tumor burden [53,54,55,56]. Taking into account all these possible pitfalls, the feasibility of liquid biopsy analyses has to be carefully investigated. Valuable lessons can be learned from comparative analyses of matched tumor tissue and plasma samples of the same patient at the same time-point. A summary of such studies is presented in Table 1.

### 3.3. Liquid Biopsies for Early Relapse Detection in Patients with Gastric and Gastroesophageal Junction Cancer

MRD detection by cfDNA analysis has been prospectively investigated in a number of studies of early stage gastric cancer being resected with curative intent [32,61,62,63]. The majority of these studies used targeted next-generation sequencing (NGS), some of which included serial measurements over the post-surgical course. An overview of these studies is shown in Table 2.

Together, these studies show that post-surgical detection of mutant cfDNA is prognostically adverse and most patients with persistent MRD eventually relapse at some point. Time to relapse appears to be generally shorter in patients with post-surgical detection of mutant cfDNA and liquid biopsy positivity typically precedes clinical relapse by several months.

The general problem of MRD detection in this group of diseases is the profound inter-patient heterogeneity of mutations. The use of mutation-agnostic MRD monitoring approaches, therefore, requires large gene panels. This is in conflict with the high sensitivity needed for MRD detection. CAPP-seq approaches and tumor-informed approaches may achieve much higher sensitivity. The latter require prior sequencing of the tumor followed by the design of patient-specific probes that can be used for highly sensitive over-time monitoring (e.g., by digital droplet PCR). Some of the limitations of tumor-informed monitoring the relatively high costs, the turn-around times for the establishment of the patient-individual assays, and the potential of losing the monitored subclone as a result of shifts in the genomic tumor landscape, e.g., on selective treatment pressure.

### 3.4. Liquid Biopsies for the Detection of Druggable Lesions in Patients with Gastric and Gastroesophageal Junction Cancer

In gastric and gastroesophageal junction cancer, there are currently two biomarkers guiding treatment decisions: ERBB2/HER2 amplification/expression for trastuzumab treatment and PD-L1 tissue expression for treatment with immune checkpoint inhibitors.

For determination of the HER2 amplification status, a genomic test is necessary, which is usually performed on DNA from formalin-fixed paraffin-embedded tissue. The amplification of ERBB2 may, however, also be done on cfDNA by a ddPCR method, which showed comparable effectiveness to immunohistochemistry and fluorescence in situ hybridization [29]. In addition to its potential use in primary genotyping of gastric and gastroesophageal junction cancer, the plasma HER2 ratio determined by ddPCR may also represent a non-invasive approach that can be used to monitor the effects of treatment in patients with HER2-positive tumors and to enable treatment options for patients with tumors that converted from HER2 negativity to HER2 positivity at recurrence without the need to obtain a new tissue biopsy [29]. Moreover, since liquid biopsies are generally better suited to capturing tumor genetic heterogeneity as described above, the use of liquid biopsies with or without additional tissue diagnostics at relapse may allow the identification of more patients for targeted treatment approaches. A large sequencing study performed on patients with gastric adenocarcinoma supported this idea. This study identified substantial heterogeneity when sequencing cfDNA, tumor, and metastatic tissue in seven confirmed HER2-positive cases [32]. Of these, only 2 (28%) showed concordant results with the 3 testing modalities. This illustrates that therapeutic decisions based on just one test or biopsy site carry the risk of missing HER2-targeting opportunities.

The U.S. Food and Drug Administration (FDA) granted Breakthrough Therapy Designation for the monoclonal antibody bemarituzumab as first-line treatment for patients with FGFR2b-overexpressing metastatic and locally advanced gastric and gastroesophageal adenocarcinoma in combination with chemotherapy. The licensing is expected to be based on an FDA-approved companion diagnostic assay showing overexpression of this target in at least 10% of tumor cells [24,25]. FGFR2 tissue expression will, therefore, become a third biomarker guiding the choice of treatment in these cancers. It is interesting to note that the recent trials studying FGFR2 targeting have included cfDNA analyses to determine the FGFR2 amplification status. In the FIGHT trial combining the FGFR-targeting antibody bemarituzumab with chemotherapy, eligible patients were selected based on FGFR2b overexpression determined by immunohistochemistry or FGFR2 gene amplification by cfDNA analysis [24,25]. Of the 155 randomized patients, only 6 could be detected as FGFR2 amplified by cfDNA analysis but not via tumor tissue testing. Another trial investigating the efficacy of the FGFR inhibitor AZD4547 [68] demonstrated high activity in FGFR2-amplified patients with gastric cancer. Copy number variation using ddPCR in tumor tissue and plasma identified all responders.

Together, this data suggests that the integration of cfDNA profiling at diagnosis or repeated profiling at relapse may help to offer a targeted treatment option to a larger number of patients with gastric and gastroesophageal junction cancer.

### 3.5. Liquid Biopsies for Disease and Resistance Monitoring in Systemic Treatment of Gastric and Gastroesophageal Junction Cancer

Finally, serial analysis of cfDNA may provide insights into the level of tumor control and the development of resistance traits in gastric and gastroesophageal junction cancers over time.

Table 2 gives an overview of key trials testing cfDNA screenings by NGS in the advanced metastatic situation and on systemic treatment [34,62,64,65,66,67,69]. Similarly to the situation in early disease after resection, serial monitoring of mutations in cfDNA identified progressive disease before clinical progression. In our own trial studying the efficacy of two experimental anti-HER2 protocols for the treatment of advanced HER2-positive gastric cancer, we found increases in cfDNA after the first treatment cycle to identify patients at risk of early treatment failure [67,69]. This makes cfDNA quantification an interesting biomarker for rapid evaluation of treatment efficacy, especially in light of the progressively broadening treatment landscape, which may enable early informed change in treatment. This is clinically relevant given that many patients with newly diagnosed metastatic gastric cancer are in need of a rapid treatment response.

Moreover, these above-mentioned trials showed that the resistance mechanisms to systemic targeted therapy are genetically heterogeneous with different routes to resistance that can even co-exist in individual patients. In HER2 targeting, the emergence of other genomic aberrations in alternative pathways, such as MYC, EGFR, FGFR2, and MET amplifications, and PIK3CA/R1/C3, ERBB2/4, and NF1 mutations may occur (refer to Table 2). In a large liquid biopsy and autopsy study, the subclonal heterogeneity of acquired resistance was studied in a patient with gastric cancer on FGFR kinase inhibition [34]. In this patient, a large spectrum of resistance subclones was detected to have heterogeneously seeded the different metastatic sites. These included cancer cells negative for the FGFR2-CD44 fusion transcript of the original main tumor clone (potential ancestors of this clone that experienced selective advantage on FGFR-targeted treatment) and clones with a variety of FGFR2 mutations within the kinase domain. Interestingly, the clones identified in the different metastatic sites on autopsy material were also found by liquid biopsy cfDNA sequencing.

## 4. Future Directions

A large body of evidence from different trials confirms that in gastric and gastroesophageal junction cancer, quantification of cfDNA can be used to assess the risk for disease recurrence or progression. A number of challenges still remain. These pertain to the sensitivity levels of detection methods, standardization of protocols, and the clinical implementation of complex tumor-informed monitoring approaches. Moreover, when predicting a lack of treatment efficacy, caution must be exercised to exclude false-positive liquid biopsy results that may result from the presence of clonal hematopoiesis or rare germline single-nucleotide polymorphisms in the patient. If these challenges can be tackled, pre- and post-surgical liquid biopsies may become a transformative technical tool to guide tailored adjuvant systemic treatment. New studies should be designed to evaluate whether adjuvant treatment intensification or drug rotation in patients with persisting liquid biopsy positivity may lead to higher cure rates.

In the metastatic setting, early drug rotation based on cfDNA dynamics may also help to reduce the percentage of patients that die early due to insufficient tumor control by their first-line treatment regimen. Admittedly, however, the numbers of active drug regimens in this spectrum of tumors are still rather limited, hampering the design of such trials.

## 5. Conclusions

Treatment personalization is geared toward boosting patient care in gastric and gastroesophageal junction cancer. Liquid biopsy testing will become increasingly relevant in this respect. In addition to their value in disease monitoring, liquid biopsies may more faithfully capture tumor heterogeneity. As a technical assay independent of tissue analysis, they may help to identify more patients for targeted treatment approaches.

Before liquid biopsies can become a standard of care in the guidance of personalized medicine in gastric and gastroesophageal junction cancer, large prospective validation trials are required.

## Figures and Tables

**Table 1:** Studies with comparative analysis of genomic profiling using liquid biopsy and matched tissue biopsy in gastric cancer.

Author	Cohort	Target	Sample Size	Result
Parikh et al., 2019 [34]	Patients with molecularly defined gastrointestinal cancers and acquired resistance to targeted therapy	Targeted NGS, multiple cancer-specific genes	23	Clinically relevant resistance alterations are more frequently identified from cfDNA
Wang et al., 2018 [57]	Patients with advanced gastric cancer before medication	HER2 amplification	56	91.1% concordance of ctDNA and tumor tissue
Schrock et al., 2018 [58]	Patients with gastrointestinal carcinomas	Hybrid capture-based genomic profiling of 62 genes	25	86% of mutations detected in tissue were also detected in matched ctDNA and, conversely, 63% of mutations found in ctDNA were also found in tissue
Pectasides et al., 2018 [59]	Patients with newly diagnosed metastatic gastric and esophageal adenocarcinomas	Mixed	28	87.5% concordance for targetable alterations in cfDNA and metastatic tissue of discordant primary and metastatic lesions
Lee et al., 2019 [60]	Patients with metastatic gastric cancer	Hybrid capture NGS of MET amplification	19	89.5% concordance rate between ctDNA and tumor, 100% concordance rate when patients without detectable ctDNA levels were excluded

**Table 2:** Key studies on liquid biopsy applications in gastric and gastroesophageal cancer *.

Cancer Entity	Technique	Conclusions	Reference
Resected GC	Targeted NGS of cfDNA	Mutant cfDNA correlates with tumor stage and post-operative positivity is prognostically adverse.	[63]
Resected GEA	Targeted NGS of cfDNA	In locoregional gastric cancer, patients treated with curative intent cfDNA-detected MRD identifies patients at high risk for recurrence	[63]
Resected and metastatic GEA	Targeted NGS of cfDNA	Post-operative MRD predicted short relapse-free survival. High mutation load at the diagnosis of metastatic disease predicted poor survival.	[61]
Resected and metastatic GEA	Targeted NGS of cfDNA	Patients with locally advanced disease and detectable mutations in cfDNA postoperatively experienced adverse outcomes. Liquid biopsies and matched tissue biopsies demonstrate significant heterogeneity and may therefore give complementary information.	[32]
Resected and metastatic GEA	Targeted NGS and WES of cfDNA	Mutant cfDNA can be detected in the plasma of GEA patients and correlates with disease burden and stage.	[62]
Locally advanced HER2+ GEA	NGS of cfDNA	cfDNA sequencing at disease progression demonstrates the emergences of other genomic aberrations, such as MYC, EGFR, FGFR2, and MET amplifications.	[64]
Mostly metastatic GEA	Targeted NGS of cfDNA	76% of patients showed mutations in cfDNA. Genomic alterations only partially overlapped with those found upon tumor tissue sequencing. Many patients had potentially druggable lesions.	[33]
Metastatic GEA treated with targeted therapy	NGS of cfDNA (WES)	The emergence of multiple resistance alterations in an individual patient may represent the ‘rule’ rather than the ‘exception’. Liquid biopsies are preferable over tissue biopsy because they better capture the heterogeneity in the setting of acquired resistance.	[34]
Metastatic HER2+ GEA	Targeted NGS of cfDNA	Serial monitoring of mutations in cfDNA identified progressive disease before clinical progression. Resistance mechanisms on HER2 targeting are genetically heterogeneous.	[65]
Metastatic HER2+ GEA	Targeted NGS of cfDNA	The study identifies PIK3CA/R1/C3, ERBB2/4, and NF1 mutations as drivers of resistance in HER2 targeting.	[66]
Metastatic HER2+ GEA	Targeted NGS of cfDNA	Early increase in cfDNA during treatment identifies individuals at risk for rapid progression. Resistance to anti-HER2 may be mediated by epitope-disrupting HER2 mutations.	[67]

* GC = gastric cancer, GEA = gastroesophageal adenocarcinoma, cfDNA = cell-free DNA, MRD = minimal residual disease, NGS = next-generation sequencing, WES = whole exome sequencing.

## Data Availability

Not applicable.

## Abbreviations

B2M	beta-2 microglobulin
BEAMing	beads, emulsion, amplification, magnetics
CAPP-seq	cancer personalized profiling by deep sequencing
cfDNA	cell-free DNA
CIN	chromosomal instability
ddPCR	digital droplet PCR
FCGR	fibroblast growth factor receptor
FDA	Food and Drug Administration
FISH	fluorescence in situ hybridization
GC	gastric cancer
GEA	gastroesophageal adenocarcinoma
Her2	human epidermal growth factor 2
IHC	immunohistochemistry staining
MRD	minimal residual disease
MSI	microsatellite instability
NGS	next-generation sequencing
RT PCR	real-time PCR
TCGA	The Cancer Genome Atlas research project
UMI	unique molecular identifyer
VEGFR2	vascular endothelial growth factor receptor-2
WES	whole exome sequencing
